# Porphyrin-Based Nanostructures for Photocatalytic Applications

**DOI:** 10.3390/nano6030051

**Published:** 2016-03-22

**Authors:** Yingzhi Chen, Aoxiang Li, Zheng-Hong Huang, Lu-Ning Wang, Feiyu Kang

**Affiliations:** 1 School of Materials Science and Engineering, University of Science and Technology Beijing, Beijing 100083, China; janechenyingzhi@163.com (Y.C.); aoxiang_li1991@163.com (A.L.); 2 Key Laboratory of Advanced Materials (MOE), School of Materials Science and Engineering, Tsinghua University, Beijing 100084, China; fykang@sz.tsinghua.edu.cn

**Keywords:** porphyrin, self-assembly, nanocrystal, nano-heterojunction, photocatalysis, solar energy conversion efficiency

## Abstract

Well-defined organic nanostructures with controllable size and morphology are increasingly exploited in optoelectronic devices. As promising building blocks, porphyrins have demonstrated great potentials in visible-light photocatalytic applications, because of their electrical, optical and catalytic properties. From this perspective, we have summarized the recent significant advances on the design and photocatalytic applications of porphyrin-based nanostructures. The rational strategies, such as texture or crystal modification and interfacial heterostructuring, are described. The applications of the porphyrin-based nanostructures in photocatalytic pollutant degradation and hydrogen evolution are presented. Finally, the ongoing challenges and opportunities for the future development of porphyrin nanostructures in high-quality nanodevices are also proposed.

## 1. Introduction

The rapid development of industrialization and civilization has posed great threats to a clean environmental set-up and sustainable energy supply. Environmental pollution is a serious day-to-day problem. Air, water and solid waste contamination due to anthropogenic sources constitute an increasing concern, to which the solution is a major challenge [1]. On the other hand, the lack of a secure long-term energy supply is also recognized as a major issue that mankind will have to face [2,3,4,5]. To address these issues, much effort has been devoted to developing strategies for environmental purification and energy generation. Since the pioneering discovery of hydrogen evolution through the photoelectrochemical (PEC) splitting of water on TiO_2_ electrodes [6], photocatalysis has been increasingly exploited for different purposes, including environmental decontamination [7,8], hydrogen generation [9,10,11,12] or organic synthesis, because of its free use of natural sunlight. Unlike the energy-intensive conventional treatment methods, photocatalysis offers many advantages and capabilities by the use of renewable and pollution-free solar energy. In the case of a photocatalysis event [13], light-harvesting and exciton diffusion take place first; then comes the charge separation, which is followed by the carrier transport. Note that first the light absorption and subsequent electron transfer construct the key factors to achieve efficient solar energy conversion for a photocatalyst. To form a photocatalyst system proficient in these specific properties and functions, the design and fabrication of nanoscale assemblies are gaining increasing attention [14,15,16].

For photofunctional materials, nanoscale architectures usually exhibit unique optical and electronic properties [17,18,19,20]. More importantly, the ability to control size and shape provides enhanced optoelectronic properties due to size- and shape-dependent effects and collective behaviors from the assembled building blocks [21,22]. So far, numerous inorganic- and organic-based nanostructures have emerged as new building blocks to construct photocatalysis systems [23,24,25,26]. However, compared to inorganic nanostructures, the organic counterparts have received special attention attributed to their considerable flexibility in molecular design, excellent tunability of the optoelectronic properties and their nice solution processability [17,27,28,29,30]. Accordingly, the investigation of the fabrication of organic nanostructures is of great importance for photocatalytic applications.

Among the various organic building blocks, the distinguished π-conjugated porphyrins are of great interest for their excellent photophysical, photochemical, electrochemical and structural properties [31,32,33,34]. The rich and extensive absorption features of porphyrins guarantee an efficient use of the solar spectrum [35,36]. Additionally, their rigid and planar molecular skeleton and inherent aromatic electronic features facilitate their assembly into well-defined nanostructures with favorable optoelectronic properties [31,37,38]. Actually, the assembly of porphyrins is characterized by their synthetic versatility and morphology controllability. Different from inorganic nanostructures, nanoscale porphyrin structures are mainly formed by self-assembly, including reprecipitation [39,40,41,42], surfactant-assisted self-assembly (SAS) [43], sonication-assisted assembly [44], vaporization, condensation-recrystallization (VCR) organization [45], ionic self-assembly [46,47], coordination polymerization [48,49,50], *etc.* Since the self-assembly of porphyrins is mainly dependent on various intermolecular noncovalent interactions (hydrogen bonding, π-π stacking, hydrophobic, electrostatic interactions and van der Waals forces), porphyrin nanostructures with a certain size, shape and function can be provided through careful molecular and supramolecular design. To date, porphyrin-based nanostructures have been extensively applied for visible-light photocatalysis [47,51,52,53]. However, the photocatalytic efficiency of the as-obtained porphyrin nanostructures is still limited by the fast recombination of photoinduced electron-hole pairs. Towards this issue, a combination of porphyrin and other optoelectronic functional materials provides routes to design new nanohybrid systems and thereby seems ideal for fulfilling enhanced light-harvesting and charge-transfer functions, which leads to a final improved solar energy conversion efficiency. In this regard, we review here the recent advances in the design and fabrication of porphyrin-based assemblies and highlight their photocatalytic applications in pollutant degradation and hydrogen generation.

## 2. Design and Photocatalytic Applications

### 2.1. Isolated Porphyrin Nanostructures for Photocatalysis

The first requirement for a photocatalyst to be applicable is light absorption in the UV-visible range. Because of the strong absorption in the region of 400–450 nm (one Soret band), as well as 500–700 nm (four Q bands), porphyrin derivatives have already been successfully used for photocatalysis over the past decades, but always in the molecular state (free or immobilized molecules) [8]. Nano-assembly takes heterogenization of porphyrins a step forward, by allowing for not only increased solar absorption due to aggregate formation, but also an enhanced stability for recycling use. It is known that organic photocatalysts are usually limited by their photostability, since they tend to undergo photobleaching or solvolysis by the solvent. Such limited stability hence incurs a gradual loss in their photocatalytic efficiency during the reaction; while this can be circumvented by nano-assembly, which provides a robust structure. The geometrical constraints imposed by the rigid aggregate framework will therefore make attack by reactive species more difficult and ensure enhanced stability for further use. In view of these merits, many papers have appeared involving the use of porphyrin nanostructures for photocatalysis.

**Figure 1:**
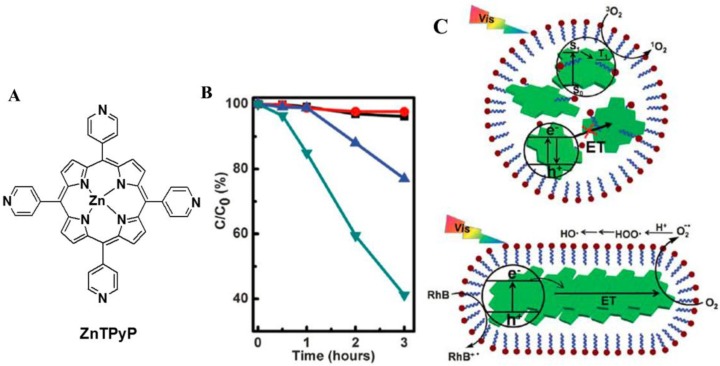
(**A**) Molecular structure of zinc-tetra (4-pyridyl) porphyrin (ZnTPyP); (**B**) photocatalytic activities of ZnTPyP-based nanospheres (●), nanospheres/nanofibers (▲), nanofibers (▼) and no catalyst (■) for rhodamine B (RhB) photodegradation under visible-light irradiation; (**C**) a schematic illustration on the structure of ZnTPyP nanospheres and nanofibers and their morphology-dependent photocatalytic performance. ET = electron transfer. Reproduced with permission from [51]. Copyright 2012, Royal Society of Chemistry.

The simplest way to produce isolated porphyrin nanostructures is reprecipitation (also called the solvent exchange method). For a porphyrin dye that is soluble in nonpolar (good) solvent, but insoluble in polar (poor) solvent, porphyrin nanostructures can be assembled through injection of a concentrated solution of porphyrin in good solvent into a pool of poor solvent; while in other cases, surfactants are added to assist in the nice control of the assembly. In one study, zinc-tetra (4-pyridyl) porphyrin (ZnTPyP; Figure 1A) nanostructures were prepared via the surfactant-assisted reprecipitation method [51]. The morphologies produced varied from nanoparticles to one dimensional (1D) nanofibers by increasing the surfactant concentration or by prolonging the aging time. Through carefully controlling the assembly behavior, ZnTPyP nanostructures exhibit specific properties and functions for morphology-dependent photocatalytic efficiency. Compared to ZnTPyP nanoparticles, 1D ZnTPyP fibers demonstrated a highly enhanced photocatalytic performance towards degrading rhodamine B (RhB) pollutants (Figure 1B). The reason for this was investigated by an electron paramagnetic resonance (EPR) analysis, which suggested an electron transfer mechanism for ZnTPyP fibers, but an energy transfer process for ZnTPyP particles, thus accounting for the morphology-dependent photocatalytic performances (Figure 1C).

**Figure 2:**
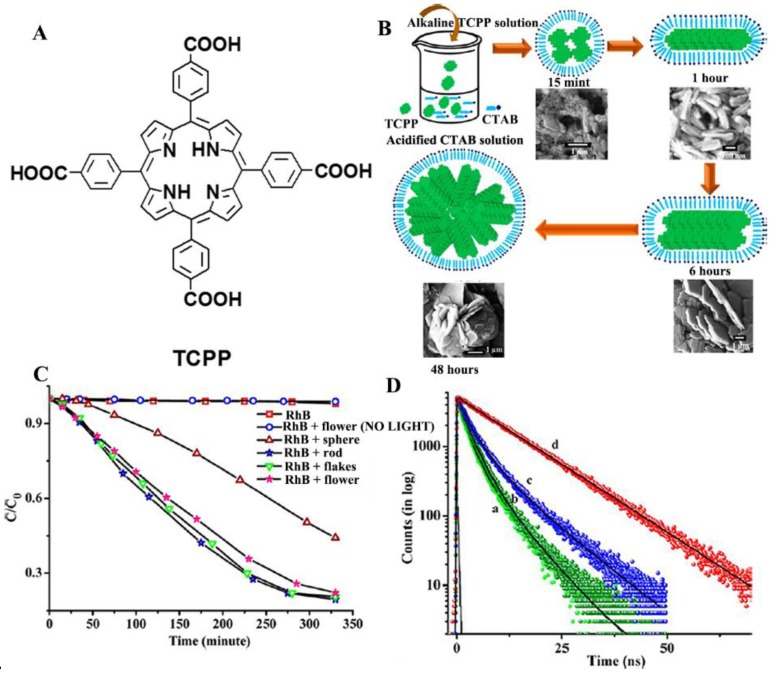
(**A**) Molecular structure of *meso*-tetra (4-carboxyphenyl) porphyrin (TCPP); (**B**) possible mechanism of the formation of different morphologies of TCPP aggregates; (**C**) photocatalytic activity of different TCPP aggregates; (**D**) photoluminescence decay curves: (a) sphere, (b) rod, (c) flower and (d) monomer. Reprinted with permission from [55]. Copyright 2014, American Chemical Society.

In fact, molecular orientation in one dimension at the nanoscale is important in boosting photocatalytic efficiency. Extensive charge delocalization within the long axes of π-π stacks and its effect on retarding the charge recombination by stabilizing the electron transfer products have been investigated in 1D organic nanostructures [54]. Further examples are the assembly of *meso*-tetra (4-carboxyphenyl) porphyrin (TCPP; Figure 2A) into different morphologies of nanostructures (spheres, rods, flakes and flowers), which are simply controlled by varying the stirring time during the acid-base neutralization process (Figure 2B) [55]. The degradation rates of RhB are calculated to be 56%, 81%, 79% and 71% for the sphere-, rod-, flake- and flower-shaped TCPP aggregates, respectively (Figure 2C). Steady-state and time-resolved spectroscopic studies (Figure 2D) reveal that the porphyrin molecules in flake- and flower-shaped aggregates are in both J- and H-aggregations, but for 1D rods, the porphyrin molecules exist in a total J-aggregation that enhances the coherent electronic delocalization for long-lived charge carriers and become a more efficient photocatalyst.

**Figure 3:**
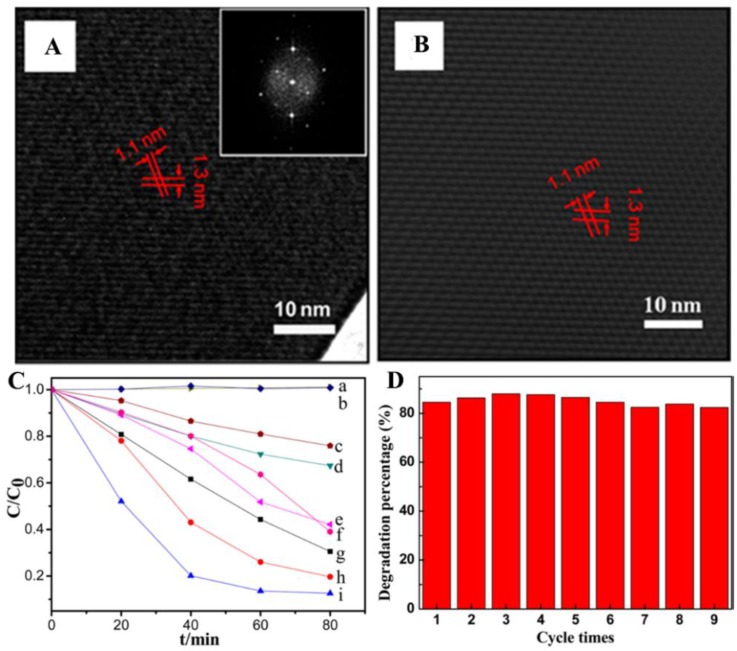
(**A**) The high resolution transmission electron microscope (HR-TEM) image of ZnTPyP tetragonal rods. The inset shows the fast Fourier transformation (FFT) of the transmission electron microscope (TEM) image. (**B**) Corresponding reverse fast Fourier transformation (RFFT) image built upon the FFT image. (**C**) Photocatalytic activities of ZnTPyP nanocrystals: no ZnTPyP nanocrystals (a), commercially available P25 TiO_2_ nanoparticles (b), tetragonal nanorods with a 200-nm length (c), same concentration of ZnTPyP in DMF (d), same concentration of ZnTPyP in 0.01 M HCl (e), nanoparticles with an 80-nm diameter (f), hexagonal nanowires with a 2-μm length (g), hexagonal rods with a 400-nm length (h) and hexagonal porous nanodiscs (i) for methyl orange (MO) photodegradation under visible-light irradiation. (**D**) Cycling tests of photocatalytic activity of ZnTPyP nanodiscs under visible-light irradiation. Reprinted with permission from [58]. Copyright 2014, American Chemical Society.

In other examples, the formation of porphyrin nanocrystals is stressed. As is known, one of the main problems associated with the use of organic nanostructures is their notoriously low charge carrier mobility, which constitutes an impediment on their photocatalytic activities. However, an increased mobility can be achieved when they are assembled into single crystals due to their perfect molecular ordering [56,57]. Experimental results have proven that higher mobility, along with higher intrinsic charge-transport properties can be observed in single-crystalline organic optoelectronic devices. In this context, it seems meaningful to synthesize photocatalytic porphyrin nanocrystals. For instance, ZnTPyP molecules (the same structure as that in Figure 1A) have been further assembled into several kinds of nanocrystals for photodegrading methyl orange (MO) [58]. The synthesis was accomplished by the self-assembly-induced micelle encapsulation method, where a known amount of acidified ZnTPyP solution was fast injected into a basic solution containing surfactants. At given pH conditions, along with increasing surfactant concentration, morphologies from amorphous nanoparticles to crystalline structures, including hexagonal nanodiscs, tetragonal nanorods and hexagonal nanorods could be obtained. For example, the morphology of ZnTPyP tetragonal nanorods is shown in Figure 3A,B. The resulting nanocrystals doubtlessly exhibited a higher photocatalytic activity than the amorphous nanoparticles (Figure 3C). Among the several crystals, porous nanodiscs showed the best performance because of their largest specific surface area. More importantly, the less defective nanocrystals encouraged a repeated use without any significant loss in efficiency (Figure 3D). Similar work was also performed on the assembly of tin porphyrin into various hierarchically-structured nanocrystals for photodegrading MO, which emphasizes, as well, the importance of forming porphyrin nanocrystals [59].

It was concluded here that a more regular molecular packing leads to a higher photocatalytic efficiency for porphyrin assemblies, and in particular, porphyrin nanocrystals perform better because their perfect molecular alignment is favorable for the enhanced electron transport process and photostability.

### 2.2. Organic p/n Nano-Heterojunction for Photocatalysis

During photocatalysis, the electron−hole charge carriers photoinduced by a photocatalyst undergo either recombination or migration to the surface of the catalyst to contribute to a series of photocatalytic reactions. To improve the catalytic efficiency, the construction of an ordered or crystalline nanostructure aiming for fewer charge trapping centers and long-lived charge carriers is one strategy (as introduced above). Besides, interfacial heterostructuring for proper energy level offsets can help increase the charge transfer efficiency greatly [60].

Similar to inorganic semiconductors, the organic counterpart has an n- or p-type character. Upon contacting an n- and p-type organic layer, the resultant energy offset provides the driving force for exciton dissociation to produce free charge carriers [61]. Over the years, organic p/n bilayers or bulk p/n heterojunctions have been recognized as a significant part of organic photovoltaic cells. Recently, photocatalysis systems featuring organic p/n bilayers or p/n nanocomposites made of phthalocyanine (closely related to porphyrins) and fullerene or a perylene derivative have been revealed for highly efficient photocatalytic hydrogen evolution (PEC water splitting) or pollutant decomposition under visible light [62,63,64].

In this study, we have succeeded in the fabrication of 1D organic single-crystal p/n nano-heterojunctions for photocatalytic degradation [65]. The crystalline junction was composed of tetraphenylporphyrin (H_2_TPP, p-type) and *N*,*N*-(dicyclohexyl) perylene-3,4,9,10-tetracarboxylic diimide (CH-PTCDI, n-type), which was prepared through a one-step physical vapor deposition (PVD) method. The crystalline nature is expected to offer better stability and charge transport properties. Furthermore, the 1D heterojunction structure possesses prominent features that are critical for efficient photocatalysis: such junctions both create a large donor/acceptor interface to separate spatial charges and also provide long-range transport pathways for charge carrier movement, thus spatially separating the photogenerated charge carriers to prevent their recombination.

**Figure 4:**
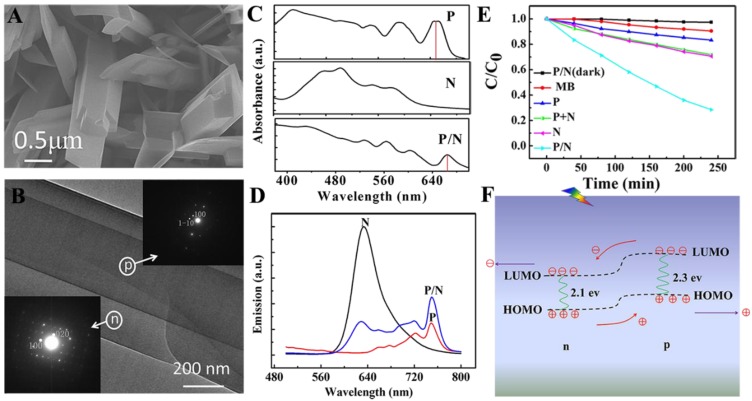
(**A**) The scanning electron microscope (SEM) image of tetraphenylporphyrin (H_2_TPP)/ N,N-(dicyclohexyl) perylene-3,4,9,10-tetracarboxylic diimide (CH-PTCDI) nano-heterojunctions; (**B**) TEM image of a single p/n nanostructure; the insets are the corresponding electron diffractions; (**C**) The UV-visible and (**D**) the fluorescence spectra of the obtained samples; (**E**) Photocatalytic degradation of MB with different samples under visible-light irradiation (λ > 400 nm); (**F**) operating principle of H_2_TPP/CH-PTCDI nanostructures. Reproduced with permission from [65]. Copyright 2013, Royal Society of Chemistry. MB, methyl blue.

SEM and TEM images proved the two-layer heterojunction morphology (Figure 4A,B). The thickness is observed to be 500 nm for the H_2_TPP layer and 200 nm for the CH-PTCDI layer in the 1D H_2_TPP/CH-PTCDI junction. The formation of this junction is strongly dependent on the similar molecular structures and close lattice parameters (10.1% mismatch) at the interfacial planes of the (020) facets of H_2_TPP and (100) facets of CH-PTCDI, which is important for epitaxial crystallization of H_2_TPP and CH-PTCDI. It thus provided an efficient p/n interface for carrier generation and charge transfer.

The absorption spectrum of the junction exhibited much broader absorption in the visible and near-infrared regions than each component (Figure 4C). The enhanced sunlight utilization will thereby be devoted to a higher solar energy conversion efficiency. Efficient electron transfer takes place at the p/n interface when excited, as proven by the remarkable fluorescence quenching of the CH-PTCDI component when hybridized with H_2_TPP (Figure 4D). The photocatalytic activities of the products have been evaluated for oxidizing methyl blue (MB) under visible light. The order of reactivity observed is as follows: H_2_TPP/CH-PTCDI junction > CH-PTCDI nanowires > H_2_TPP + CH-PTCDI mixture > H_2_TPP nanoplates (Figure 4E). The higher efficiency of the H_2_TPP/CH-PTCDI junction is attributed to increased light absorption and the wide p/n interfaces available for charge separation. The detailed operating principles are illustrated in Figure 4F. Light absorption takes place at the p side, with the result of exciton formation and subsequent diffusion to the p/n interface at which the exciton readily dissociates. Then, the separated electrons are transported away rapidly through the 1D crystalline n-CH-PTCDI side, while the left holes flow to the p-H_2_TPP side to oxidize MB.

### 2.3. Organic/Inorganic Nano-Heterojunction for Photocatalysis

Let us focus on the organic/metal nanohybrid first. Metallic nanoparticles exhibit some interesting catalytic, optical and electric properties, which can help improve the photocatalytic activity. In particular, concerning photocatalytic hydrogen evolution, precious-metal species (Pt or Au) are always involved as cocatalysts, which help to promote the photoinduced charge transfer from porphyrin nanostructures to the Pt surface at which water is converted to hydrogen gas. Nevertheless, this is still exciting, because porphyrin nanostructures are able to use visible light to photocatalytically grow metal nanoparticles onto their surfaces in the presence of an appropriate metal complex and a sacrificial electron donor. For Pt complexes, the reactions are as follows:

P=Porphyrin, ED=electrondonorP+hv→Pt0P*P*+ED→Pt0P•−+EDox2P•−+Pt2+→Pt02P+Pt0

Based on this strategy, self-platinized porphyrin nanotubes, nanosheets and nanofiber bundles have been successively synthesized by Shelnutt *et al.* [42]. Indeed, the porphyrin/Pt nanohybrid can be used for photocatalytic hydrogen evolution [47]. When visible light along with an electron donor, like ascorbic acid or triethanolamine, are present, the platinized porphyrin nanostructures evolve hydrogen by the reaction:

P+hv→Pt0P*P*+ED→Pt0P•−+EDox2P•−+2H+→Pt02P+H2

**Figure 5:**
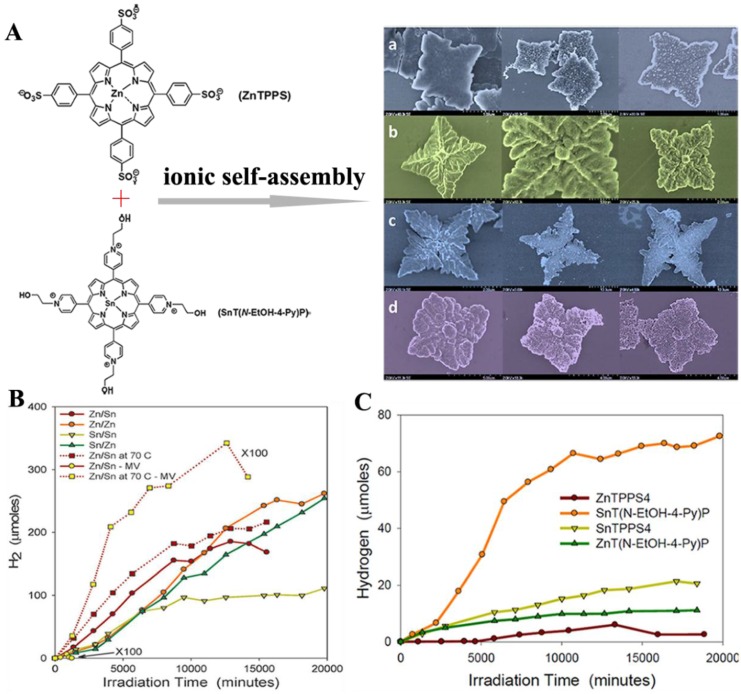
(**A**) SEM images of the structures prepared at room temperature for all four combinations of Zn(II) and Sn(IV) in the two porphyrins (left), the corresponding unwashed platinized clovers (middle) and the washed clovers after two weeks of continuous hydrogen generation (right): Zn/Sn (a), Sn/Zn (b), Zn/Zn (c) and Sn/Sn (d) by ionic self-assembly of Zn(II) tetrakis(4-sulfonatophenyl)porphyrin (ZnTPPS) and Sn(IV) tetrakis(*N*-2-hydroxyethyl-4-pyridinium)porphyrin (SnT(*N*-EtOH-4-Py)P); (**B**) Total H_2_ generated by the platinized porphyrin clovers (Zn/Sn, Zn/Zn, Sn/Sn, Sn/Zn) grown at room temperature (solid lines) and at 70 °C for the Zn/Sn clovers (dotted lines) *versus* irradiation time with visible light from a tungsten lamp. Zn/Sn clovers with (dark red symbols) and without (yellow) methylviologen. The latter are expanded by a factor of 100; (**C**) Hydrogen generated by free porphyrins at the same concentrations as in the clovers as a function of irradiation time by white light at 0.15 W·cm^−2^. Reproduced with permission from [66]. Copyright 2011, Royal Society of Chemistry.

Self-metallization is also applicable to binary porphyrin structures for hydrogen evolution [66]. These complex structures are ionically self-assembled by taking advantage of the electrostatic interactions between anionic and cationic porphyrins. The oppositely-charged pairs admit an enhanced photoinduced electron transfer across the porphyrin subunits, leading to higher photoactivity than individual porphyrins [67]. For instance, Shelnutt *et al.* reported the four-leaf clover-like morphologies by ionic self-assembly of different Zn (II)- and Sn (IV)-based porphyrin pairs (Figure 5A) [66]. Self-platinization of these porphyrin clovers was done by exposing them to a mixing solution of K_2_PtCl_4_ and ascorbic acid solution under visible-light irradiation (0.1 W·cm^−2^). Figure 5B compares the H_2_ production activities exhibited by different platinized Zn and Sn porphyrin pairs. Hydrogen generated by free porphyrins is exhibited in Figure 5C. Of these catalysts, clovers show a strikingly increased activity compared to individual porphyrin structures. It should also be noticed that the performance of the clovers is better than the sum of their individual effects, which can be explained by the cooperative interaction within the binary solids. Certainly, different ionic and cationic combinations cause a difference in the H_2_ generation rate that can mainly be ascribed to their varied cooperative interaction.

In terms of electric properties, metallic nanoparticles can store charges and promote charge transfer reactions. Because of the participation of gold nanoparticles, an increase in charge separation distance has been shown in thin film assemblies of porphyrin-fullerene dyads through the enhanced charge transfer from porphyrins to gold [68,69]. The interesting optical characteristic of metallic nanoparticles is the surface plasmon resonance (SPR). Plasmonic metal nanoparticles have been extensively embedded on inorganic nanostructures for enhanced photocatalytic activity [70]. On the other hand, the influence on the electronic excitation of porphyrin molecules by plasmonic metals has contributed to a large enhancement of photocurrent signals, as reported [71,72]. Gold nanoparticles with diameters of several nanometers show distinct SPR absorption bands in the visible to near-infrared region. Thus, porphyrin-gold nanoparticle multistructures are expected to enhance the electronic excitation of porphyrin molecules by the SPR effect, leading to enhanced electron transfer events, more efficiently in the far-red to near-infrared region. Yet, the plasmonic enhancement of photocatalysis has mostly worked on the molecular porphyrins, and it is necessary to make further efforts with porphyrin/metal nanohybrids for plasmonic photocatalysis.

**Figure 6:**
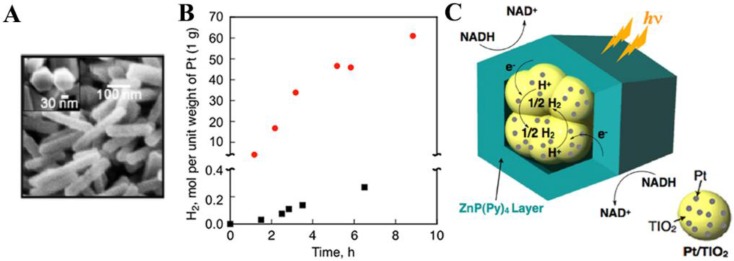
(**A**) SEM image of Pt/TiO_2_-ZnP(Py)_4_ nanorods; (**B**) Time dependence of hydrogen evolution: (●) Pt/TiO_2_-ZnP(Py)_4_ rods, (■) non-encapsulated Pt/TiO_2_ + ZnP(Py)_4_ composites. (**C**) A schematic illustration for photocatalytic hydrogen evolution. Reprinted with permission from [75]. Copyright 2013, American Chemical Society.

An alternative strategy to construct heterogeneous interfaces is to design organic/metal oxide nano-heterojunctions. Such junctions can take advantage of the broad sunlight absorption of the organic component and the high charge carrier mobility of the inorganic semiconductor counterpart [73]. Porphyrin-TiO_2_ core-shell nanoparticles have been successfully synthesized using a sol-gel reaction [74]. The composites combine the desirable properties of both organic and inorganic components and exhibit better MB photodegradation compared to other porphyrin/TiO_2_-based photocatalysts. Another interesting example was provided by using porphyrin hexagonal nanocylinders that encapsulate Pt/TiO_2_ nanoparticles in the internal cavity for photocatalytic hydrogen evolution [75]. The final three-component structure was denoted as Pt/TiO_2_-ZnP(Py)_4_ nanorods, which were prepared by a surfactant-assisted reprecipitation method (Figure 6A). Figure 6B indicates the very efficient hydrogen generation achieved by Pt/TiO_2_-ZnP(Py)_4_ nanorods under visible-light irradiation, much better than the non-encapsulated Pt/TiO_2_ + ZnP(Py)_4_ mixture. Hydrogen evolution may be initiated by photoinduced electron injection from the porphyrin singlet excited state (ZnP(Py)_4_^•+^/^1^ZnP-(Py)_4_* = −1.09 V *vs.* SCE) to the conduction band of TiO_2_ (~−0.7 V *vs*. SCE) in Pt/TiO_2_-ZnP(Py)_4_ nanorods, as illustrated in Figure 6C. The injected electrons migrate toward Pt nanoparticles on the TiO_2_ surface to reduce H^+^ to H_2_.

### 2.4. Others

Regarding the characteristic π-conjugated structure, carbon nanotubes, graphenes or C_3_N_4_ can also be taken into account as an ideal network to promote charge transfer and electron transport from porphyrins to their collecting surface [76,77,78,79]. Thus far, significant efforts have been made to link porphyrin molecules with them via covalent and noncovalent interactions (e.g., π-π stacking, van der Waals and/or electrostatic interactions) [80,81,82,83]. A logical step forward would be to anchor porphyrin nano-assemblies on them by increasing the loading content and stability.

Graphene sheets have a particularly high theoretical surface area of ~2600 m^2^·g^−6^, making them highly desirable as a 2D catalyst support for the increased loading content of porphyrin assemblies [84,85,86]. In addition, the strong affinity of electron-donating porphyrins toward electron-accepting graphene films facilitates a charge-transfer interaction, and then, the separated electrons can be transported away through highly conductive graphene to delay the instant recombination of electron-hole pairs. To date, several attempts have been made to improve the photocatalysis by taking advantage of porphyrin/graphene nanohybrids.

**Figure 7:**
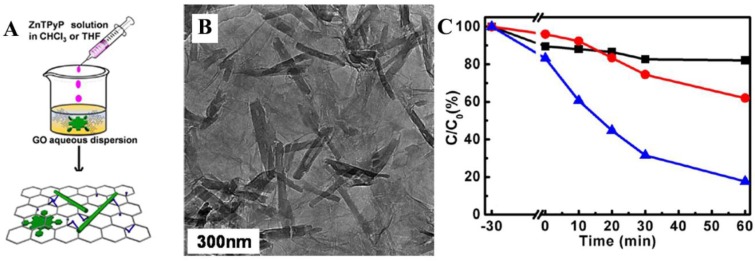
(**A**) Schematic illustration for the assembly of ZnTPyP 1D nanostructures via graphene oxide (GO)-based surfactant-assisted self-assembly (SAS); (**B**) TEM image of ZnTPyP/GO complexes; (**C**) photocatalytic activity of the original GO nanosheets (black ■), 1D ZnTPyP nano-assemblies formulated via the CTAB-assisted (red ●) and GO-assisted (blue ▲) SAS, for the photodegradation of RhB under visible-light irradiation. Reprinted with permission from [53]. Copyright 2013, American Chemical Society.

In one case, Liu *et al.* have reported the complexation of 1D ZnTPyP nano-assemblies with 2D graphene oxide (GO) via an SAS method [53]. The assembly was performed by injection of a solution of ZnTPyP in chloroform into an aqueous dispersion of GO (Figure 7A). Here, GO nanosheets could play the role of a sheet-like surfactant in the dispersion to assist ZnTPyP assembly. As a result, 1D ZnTPyP nanostructures with a diameter of *ca*. 40–60 nm and a length of *ca.* 200–300 nm were observed to form on the gauzelike GO nanosheets (Figure 7B). Figure 7C compares the photocatalytic activities for degrading RhB by GO-assisted and CTAB-assisted ZnTPyP nano-assemblies. The higher degradation rate seen with ZnTPyP/GO complexes than with CTAB-assisted ZnTPyP assemblies highlights the beneficial role of graphene sheets. Electrochemical impedance spectral (EIS) analysis indicates that charge transfer resistance of the GO-assisted ZnTPyP nano-assemblies is distinctly smaller than that of CTAB-assisted assemblies. Moreover, the G-band of GO shifts by 11 cm^−1^ to a lower frequency in ZnTPyP/GO complexes, shown by Raman spectra, confirming the occurrence of charge transfer between the GO and the ZnTPyP moieties. All indicate that the presence of GO nanosheets can promote the charge transfer in ZnTPyP/GO complexes.

**Figure 8:**
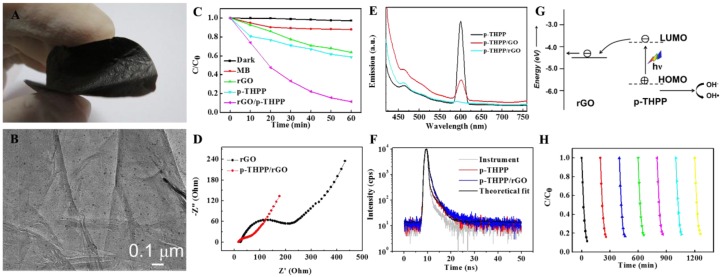
(**A**) The optical and (**B**) TEM images of free-standing meso-tetra (p-hydroxyphenyl) porphyrin (p-THPP)/rGO films; (**C**) photocatalytic degradation of MB with different samples under visible-light irradiation (λ > 400 nm); (**D**) Nyquist plots collected by EIS of free-standing rGO and p-THPP/rGO films; (**E**) the fluorescence spectra of the synthesized films; (**F**) the fluorescence decay profiles of p-THPP and p-THPP/rGO nanohybrid in H_2_O (λ_exc_ = 405 nm); (**G**) proposed mechanism for the photocatalysis of the p-THPP/rGO film; (**H**) recycling experiment using the p-THPP/rGO for MB degradation under visible-light irradiation (λ > 400 nm). Reproduced with permission from [92]. Copyright 2014, Royal Society of Chemistry.

Graphenes have also shown favorable flexibility and, hence, are promising for producing flexible and bendable free-standing scaffolds [87,88,89,90,91]. Our recent work on the photocatalytic degradation of RhB and MB highlights the role of free-standing porphyrin/graphene nanohybrid film in improving photocatalytic performance, as well as its practical convenience [92]. The formation of this free-standing film was realized by the flow-directed assembly of the stable aqueous co-colloids of graphene oxide (GO) sheets and meso-tetra (p-hydroxyphenyl) porphyrin (p-THPP) nanoparticles followed by gaseous reduction. Because of the similar π-conjugated and flexible structure, the resultant p-THPP/rGO nanohybrid films retained good integrity and flexibility (Figure 8A,B). The Soret band and Q-bands of p-THPP in p-THPP/rGO nanohybrids are all observed to red shift to some extent, compared to individual p-THPP nanoparticles, indicative of a charge transfer process.

When we compare the improved photocatalytic performance of the p-THPP/rGO film, a two-fold enhancement is observed in the photocatalytic degradation rate (Figure 8C). EIS results showed a much lower charge transfer resistance (*R*_ct_ ≈ 46.7 Ω) for p-THPP/rGO film than that for rGO film (*R*_ct_ ≈ 176.2 Ω) upon photoirradiation (Figure 8D). Such photoresponsivity is attributed to the presence of large donor/acceptor interfaces that induce charge separation. The emission of excited p-THPP can also serve as a probe to monitor the interaction between p-THPP and GO or rGO in the corresponding nanohybrid. The quenching of the emission band at 600 nm by GO or rGO thus corresponds to an interfacial electron-transfer process in the hybrid (Figure 8E). The electron-transfer process was independently confirmed by monitoring the emission lifetime. An increase in the average lifetime of p-THPP emission from *ca*. 362–473 ps was observed when hybridized with rGO (Figure 8F). The prolonged lifetime implies the excited electron-transfer process. Electron injection from the excited p-THPP to the rGO takes place, given that the LUMO band of p-THPP is ~0.9 V *vs*. normal hydrogen electrode (NHE), and the Fermi level of rGO is ~0 V *vs*. NHE. Such a difference in the energy levels enables the electron transfer from the excited porphyrin to rGO, giving evidence that rGO can work as a supportive scaffold to collect and transport photogenerated electrons, which is finally responsible for the high photodegradation rate (Figure 8G). More importantly, the monolithic nature makes the recycling use of p-THPP/rGO film more convenient. The recycling experiment (Figure 8H) showed that photocatalytic efficiency displayed only a slight decrease when conducted seven times consecutively. Furthermore, the easy separation of the monolithic photocatalyst from the reaction medium promotes its practical utility to eliminate the organic pollutants from wastewater.

**Figure 9:**
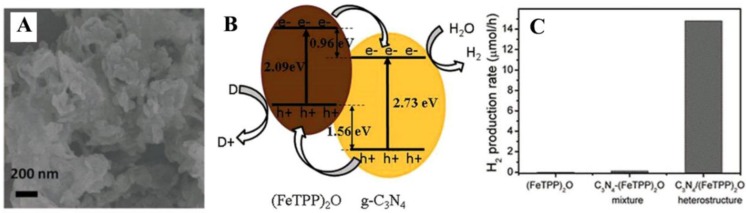
(**A**) SEM image of the graphitic C_3_N_4_ (g-C_3_N_4_)/m-oxo dimeric iron (III) porphyrin ((FeTPP)_2_O) heterostructure including 5 wt% (FeTPP)_2_O; (**B**) The band-structure diagram of the g-C_3_N_4_/(FeTPP)_2_O heterostructure; (**C**) photocatalytic H_2_ production rates over pure (FeTPP)_2_O, the g-C_3_N_4_ + (FeTPP)_2_O (5 wt%) mixture and g-C_3_N_4_/(FeTPP)_2_O (5 wt%). Reproduced with permission from [97]. Copyright 2016, Royal Society of Chemistry.

Graphitic C_3_N_4_ (g-C_3_N_4_) is another 2D framework of tri-s-triazine connected via tertiary amines, which makes it possess high thermal and chemical stability (against acid, base and organic solvents). This polymer-like semiconductor with a band gap of ~2.7 eV can function as a metal-free photocatalyst for the extraction of hydrogen from water [93,94,95,96]. The band match between porphyrins and g-C_3_N_4_ makes a combination of them available for enhanced visible-light photocatalytic hydrogen evolution in the absence of a cocatalyst. In one case, through a simple liquid chemical reaction between g-C_3_N_4_ and the precursor of m-oxo dimeric iron (III) porphyrin ((FeTPP)_2_O), a heterostructure of g-C_3_N_4_/(FeTPP)_2_O was constructed on the basis of the π-π and the Fe-amine interactions (Figure 9A) [97]. The g-C_3_N_4_/(FeTPP)_2_O nanocomposites show the absorption features combining g-C_3_N_4_ and (FeTPP)_2_O, with an obvious red shift of the Q band of (FeTPP)_2_O in the composites for increased solar utilization. Quenching of fluorescence of (FeTPP)_2_O by g-C_3_N_4_ in the composites is readily indicative of the electron-transfer process between the two moieties, which is ascertained from the band-structure diagram of the g-C_3_N_4_/(FeTPP)_2_O heterostructure (Figure 9B). As a result, the obtained g-C_3_N_4_/(FeTPP)_2_O heterostructure has displayed dramatically improved photocatalytic hydrogen production under solar light without any cocatalysts, compared to pure or mixed g-C_3_N_4_ and/or (FeTPP)_2_O (Figure 9C).

## 3. Summary and Outlook

To summarize, this paper has outlined the recent significant advances related to porphyrin-based nanostructures for photocatalytic pollutant oxidation or hydrogen evolution under visible-light irradiation. Organizing porphyrin nanostructures can benefit significantly from the improved photostability and light-harvesting property based on aggregation. An increased charge transfer/transport process can be achieved by texture or crystal modification and interfacial heterostructuring for final enhanced photocatalytic performance. The unique structure and tunable functionalization of porphyrins render their assembly or heterostructuring easily tailored through various valent or non-valent interactions, so as to better activate the interface or surface for charge transfer. This is a clear advantage when compared to inorganic photocatalysts. On this basis, significant progress has been achieved, yet extended efforts are still required in various aspects to further advance the utilization of porphyrin-based nanostructures for visible-light photocatalysis.

Among the issues requiring further efforts, the first issue is how to bring together two different types of organic and inorganic semiconductors with a large and beneficial interface. Moreover, because the electron transport through inorganic materials is very efficient, the overall electron flow in such junctions is mostly determined by the interface itself or by the lower mobility of the organic component. It thereby requires continued efforts to find better ways for improving the charge mobility of porphyrin nanostructures. Further exploration is still needed on porphyrin/metal nanohybrids for plasmonic enhancement of photocatalytic efficiency. To gain a significant breakthrough in photocatalytic performance, an in-depth understanding of interface geometry, electronic properties, excitation dynamics and charge transport properties by rational calculation and simulation would be useful for the design of efficient porphyrin nanostructure photocatalysts. Alongside the heterogeneous photocatalysis, transferring or growing porphyrin-based nanostructures onto an electrode by a proper deposition method is desired for PEC water-splitting hydrogen evolution. Photoelectrodes have the advantage that an electric field can be created at the photoelectrode/electrolyte junction to manipulate the charge transfer reaction. Particularly in the case of flexible PEC devices, the high flexibility of the porphyrin structure and its compatibility with flexible and lightweight substrates may open broader applications for the next generation of optoelectronic devices. Future improvement of energy conversion efficiency calls for an effective alignment of these molecular assemblies at the macroscopic level aiming for a better control on directional charge movement, as well as light absorption pathways in the nanostructure arrays.
